# Psychometric properties of the Chinese version of the contesting orientations scale among Chinese undergraduates

**DOI:** 10.3389/fpsyg.2025.1504580

**Published:** 2025-05-14

**Authors:** Tianxia Chen, Wanlin Li, Yilin Ren, Huiqin Zhang, Fengshu Zhu

**Affiliations:** ^1^College of Physical Education, Yangzhou University, Yangzhou, Jiangsu, China; ^2^Department of Education, Zhanjiang University of Science and Technology, Zhanjiang, China; ^3^College of Sports, Zhuhai Research Center for Women and Children's Sports Culture, Jinan University Zhuhai Campus, Zhuhai, Guangdong, China; ^4^Laboratory and Equipment Management Office, Yangzhou University, Yangzhou, Jiangsu, China

**Keywords:** contesting orientations, partnership orientation, war orientation, reliability, validity

## Abstract

**Objective:**

To modify the Contesting Orientations Scale (COS) and test its internal consistency, stability over time, construct validity, and convergent validity in conjunction with the Chinese context.

**Methods:**

A preliminary COS test was conducted on 120 college athletes nationwide (116 valid questionnaires were collected). Then, project analysis and exploratory factor analysis were used to determine a formal scale. Finally, 235 college athletes were selected for formal testing (227 valid questionnaires were recovered), and confirmatory factor analysis and internal reliability test were conducted on them; In addition, this study also used the Competitive Psychology Scale for College Students (PPCSCS) as the research object for the convergent validity of standards, and studied the correlation between competitive psychology and competitive orientation. After 1 month, 150 subjects were selected for retesting.

**Results:**

The Chinese version of COS contains 12 entries, including two dimensions: partnership and war; The confirmatory factor analysis results show that the scale has good structural validity (χ^2^ = 108.67, *df* =53, RMSEA = 0.07, NFI = 0.91, CFI = 0.95, GFI=0.93, PGFI = 0.63), The correlation coefficient between the scores of various dimensions of COS and the total score of PPCSCS ranges from 0.61 to 0.81 (*P* < 0.01). The total internal consistency coefficient of the scale is 0.79, and the retest reliability is 0.78. The internal consistency of the two dimensions of partnership and war are 0.85 and 0.88, respectively. After deleting a certain entry, Cronbach's α was not found. The significant change is that the discrimination between the various items of the scale is good, with a Guttman half reliability coefficient of 0.71 and retest reliability of 0.83 and 0.86, respectively.

**Conclusion:**

Through the competition orientation scale tested in the group of college athletes in Jiangsu Province, China, there are good measurement indicators in the selected sample of college athletes, which can predict the competition orientation of college athletes in Jiangsu Province, China and their sports personality. Future research should expand the sampling range, and sample from multiple regions and age groups to test the applicability of the Chinese version of COS, and promote it on a larger scale.

## 1 Introduction

Sports are about competition with others, and the core competitive drive of the sports experience is the main reason why some people highly value and enjoy sports, while others neither appreciate nor enjoy sports. The tendency to compete in sports is influenced by dysfunctional stress, high anxiety, unstable self-esteem, interpersonal hostility, etc. (Kohn, 1992). Classic competition theorists such as Johnson have emphasized the goal structure of competition. According to this view, there is a direct relationship between the goal structure of a competition and the internal goal pursuits of participants, which is related to the adversarial and zero-sum nature of the “competition” structure (Johnson and Johnson, 1989). Based on long-term follow-up research on “competition,” Shields et al. (2015). Proposed a newer theoretical paradigm—the form of competition theory. Competition theory believes that the experience and implementation of competition are not only affected by the environment, but also by the factors that support the participants' meaning construction process. The influence of metaphor in the concept of implicitness, different conceptual metaphors produce different competition orientations. In addition, these implicit metaphors have moral significance because they imply different social and moral relationships among participants, and different competing tendencies are likely to have different moral frameworks and foundations.

But most previous research on “competition” has focused on how the goal structure of a competition affects participants (Deutsch, 1949; e.g., Social interdependence theory posits that important outcomes, such as productivity, satisfaction, and ethical behavior, are shaped by the types of goal interdependencies that exist in situations). This approach ignored the constructive process of participant initiative. Based on competition theory, competition leads to an understanding of fair competition as a relationship between participants, who regard competition as an opportunity to work hard and progress with their common opponents, making the competition process more consistent with their requirements. In the original meaning, opponents at the same time are understood as common participants of value and necessary confrontation in the process of joint pursuit of excellence; another type of equal competition is understood as war, where participants regard competition as a kind, while opponents are understood as competing with each other. Enemies between targets. In this situation of partnership metaphor, participants will pay more attention to self-development and improve their abilities through opponents, which will bring positive competitive effects; in the situation of war metaphor, participants will be win-oriented, win again and be afraid of losing. They may even adopt some unethical means such as aggressive behavior to win the game, thus bringing about vicious competition. Other studies have shown that competitive orientation has a significant predictive effect on participants' perseverance and self-control (Shields et al., 2018). At the same time, competitive orientation has a good incremental predictive effect. Among the various assessment dimensions of participants' personality characteristics, competitive orientation is higher than the role of goal orientation, empathy, autonomous motivation, controlled motivation, moral identity and moral detachment. Of the two controversial orientations, the partnership orientation appears to be closer to the formalist orientation. First, both the partnership metaphor and the formalism emphasize the need for reciprocity, equality, and mutual respect, which are conceptually consistent. Furthermore, the relationship between partnership and war metaphors is similar to the relationship between formalism and consequentialism in that the former incorporates a greater degree of cognitive complexity in each pair. The war orientation is derived in a fairly direct way from the competition's hostile goal structure. In contrast, a partnership orientation considers antagonistic goal structures while integrating them into broader goals and social relationships. Understanding the fundamentally cooperative nature of true competition, which a partnership approach requires, requires reconciling the self-interested pursuit of victory with the underlying pleasure and mutual benefit of the mutual process (Shields and Bredemeier, 2011). However, when war metaphor thinking is activated, the situation is different. In the context of competition, especially in team-based competitions, team cohesion and unity are central issues, and success often depends on individuals subordinating their own interests to the common good of the group, the in-group/out-group delimitation of the war metaphor Bring these concerns to the fore. In contrast, war tendencies appear to be based on ideas and values inherent in the foundation of constraints, viewing the opponent as an obstacle to success. Moral categorizations of the kind described by Bredemeier and Shields (1984, 1986) may emerge more broadly when athletes conceptualize competition through war metaphors. This explanation is supported by at least three observations: (a) Partnership is more consistent with all tested moral considerations than war; thus, when the preference for partnership underpins one's competitive experience, greater emphasis will be placed on the moral standards of the game and relationships with others; (b) Formalism predicts partnership, not war, and formalism is arguably a more advanced moral framework; therefore, employing reasoning similar to “low-stage” moral thinking may be possible for those who subscribe to the partnership metaphor hard to accept; (c) Conversely, those who prefer war metaphors show a stronger relationship with consequentialist than formalist thinking, which, according to Reynolds (2006), reflects lower levels of moral cognitive sophistication. In Chinese culture, concepts such as “meeting friends through competitions” are highly compatible with the Confucian idea of “harmony is precious,” and Chinese athletes may be more inclined to see their opponents as partners for common progress (Tang et al., 2023). Athletes have long been influenced by collectivist honors such as “winning glory for the country,” and the war orientation of Chinese athletes may show the characteristics of “fighting for the team” rather than “defeating the opponent for the individual” (Zhang et al., 2021). Although there are currently measurement tools on competitive orientation around the world, there is a lack of competitive orientation measurement tools developed specifically for athletes. For example, the Chinese Cooperation and Competition Orientation Scale compares the competitive orientation of Chinese and Western college students and company employees, and is suitable for measuring competitive orientation in an environment with strong organizational relationships (Chen et al., 2011); another example is the Multidimensional Competitive Orientation Scale (Multidimensional Competitive Orientation Scale, MCOI) used subjects who were college students from a certain university and members of the Internet group (Gábor et al., 2018). There are also some measurement scales about athletes' sports competition, which are mostly at the athlete motivation level (Chen and Yin, 2008), coping style level (Cui and Zhang, 2014), etc., all of which ignore how sports participants actually understand the nature of competition and the differences between different participants. Therefore, it is necessary to revise the scale with more targeted and better measurement properties.

In the fields of psychology and sports, athletes' competitive orientation has received widespread attention. The competitive orientation scale compiled by Shields et al. is based on competition theory (Shields et al., 2015), achievement motivation theory (Nicholls, 1984), Martens' four stages of competition (Martens, 1975), recognition. The theory of cognitive linguistics (Lakoff, 2009) starts from how sports participants actually understand the basic nature of competition and the differences in understanding between different participants. What is shown is the way of thinking of athletes. This scale has good reliability and validity among American intercollegiate college athletes, and has a predictive effect on the sports personality of college athletes. Since there is currently no Chinese version, this article attempts to modify the Chinese version and verify its reliability and validity. degree to provide a new and reference measurement tool for the development of sports personality of Chinese college athletes.

## 2 Methods

### 2.1 Translation

Before making any modifications, it is essential to first contact the authors of the scale and obtain their consent. Subsequently, two students specializing in sports psychology will carry out the English translation. Based on their translations, a preliminary Chinese version of the scale will be developed. Following this, we will invite a leading professor in the field of English to perform a back-translation of the scale, which will then be revised accordingly. The revised version will be translated back into Chinese and compared with the original English version. Finally, we will invite two experts in sports psychology to evaluate the scale, confirming its various indicators and scoring methods. An expert panel consisting of four specialists will use a five-point Likert scale, with scores ranging from “1 to 5” representing “not at all relevant,” “slightly relevant,” “relevant,” “strongly relevant,” and “very relevant” in that order. Through the evaluation of each item on the scale, we will calculate its content validity and, in conjunction with the experts' feedback, revise the Chinese version of the Competitive Orientation Scale (COS).

### 2.2 Participants

Sample 1 (Prediction Sample, used for item analysis and exploratory factor analysis): we selected 6 universities in Jiangsu Province and conducted a survey on 120 college athletes using a simple convenience sampling method. Of the 120 athletes, 67% were men and 33% were women; Basketball accounted for 21%, football accounted for 16%, track and field accounted for 19%, table tennis accounted for 10%, traditional sports accounted for 12%, swimming accounted for 8%, volleyball accounted for 14%; 26% are freshmen, 27% are sophomores, 31% are juniors, and 16% are seniors. We distributed questionnaires through Questionnaire Star.

Sample 2 (Formal Testing Sample, used for confirmatory factor analysis and internal consistency analysis): we selected 12 universities (Including all 6 from Sample 1 plus 6 newly added universities from previously underrepresented provinces, the 12 institutions in Sample 2 include all 6 institutions in Sample 1, and the new institutions are all from cities not covered by the first sample.) in Jiangsu Province and used simple convenience sampling to select 235 college athletes, of the 235 athletes, 64% were men and 36% were women; Basketball accounted for 22%, football accounted for 19%, track and field accounted for 16%, table tennis accounted for 11%, traditional sports accounted for 11%, swimming accounted for 8%, volleyball accounted for 13%; 24% are freshmen, 28% are sophomores, 33% are juniors, and 15% are seniors. One month later, 150 people in sample 2 were selected for retesting to test the test-retest reliability, and the questionnaire was distributed through Questionnaire Star.

### 2.3 Measures

#### 2.3.1 Contesting orientations scale

The scale was compiled by Shields et al. (2015). It includes two dimensions: partnership metaphor and war metaphor, with a total of 16 items. It uses the Likert 5-point scoring method (1 = strongly disagree, 2 = disagree, 3 = indifferent, 4 = agree, 5 = strongly agree). The higher the score, the more likely the individual is to choose one of the competitive orientations.

#### 2.3.2 Psychometric properties of competitiveness scale of college students

We used the Psychometric Properties of Competitiveness Scale of College Students (PPCSCS) as the standard scale. In this scale, competitive psychology consists of four dimensions: competitive tendency, competitive motivation, competitive strategy and competitive content. It uses the Likert 5-point scoring method (1 = strongly disagree, 2 = disagree, 3 = indifferent, 4 = agree, 5 = strongly agree). The Cronbach's α coefficient of the college students' competitive psychology scale in this study is 0.90.

### 2.4 Data analysis

SPSS 25.0 was used to conduct item analysis, exploratory factor analysis, internal consistency coefficient, split-half reliability, test-retest reliability, and calibration scale correlation validity test, and Amos 26.0 was used to conduct confirmatory factor analysis on the scale.

## 3 Results

### 3.1 Item analysis

We conducted item analysis on the data of prediction sample and conducted correlation tests on the 12 items. We found that the total correlation coefficients of the 12 items were all >0.30, and the correlation coefficients between each item and the total score of the scale were between 0.36 and 0.73 (all *P* < 0.001). The results showed that all 12 items met the retention conditions and were not eliminated in the item analysis.

### 3.2 Exploratory factor analysis

**Figure 1:**
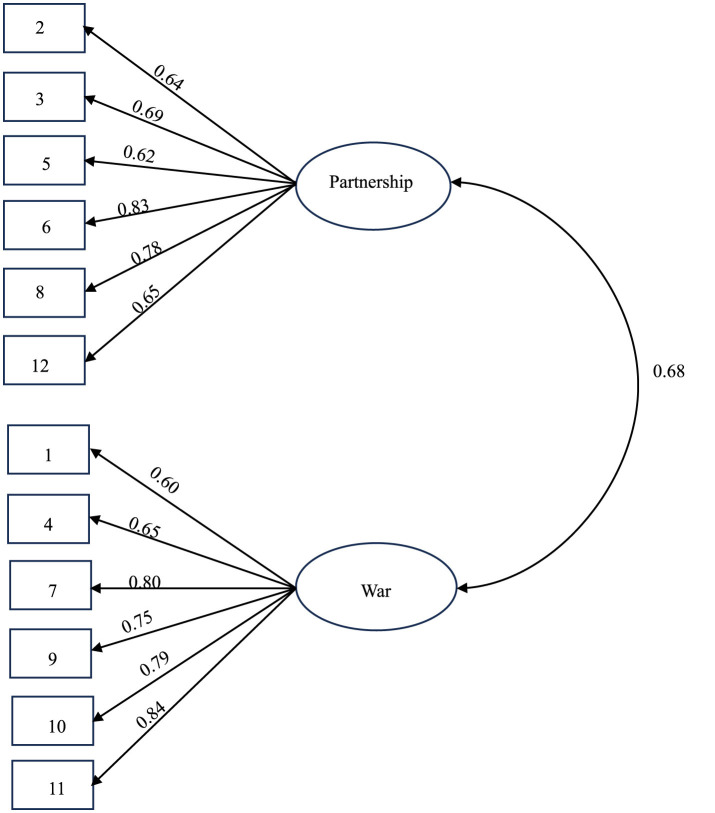
Confirmatory factor analysis of model fit.

By using principal component analysis (PCA), given the study's focus on variance maximization (rather than latent structure identification) and the moderate sample size, PCA was deemed more appropriate due to its stability in such conditions (Fabrigar et al., 1999), Promax oblique rotation and other methods on 12 items, we obtained a KMO value of 0.84, and Bartlett's spherical test reached the significance level (χ^2^ = 1,232.45, *P* < 0.001), indicating that the research can continue. Through comprehensive analysis of the scree plot, two main control factors were obtained. The number of items in each factor is 3 or above, which meets the structural stability requirements of the scale. Based on the research results, a 12-item 2-dimensional competitive orientation scale was compiled. Referring to the original English version of COS, David et al. classified these two factors as “war” and “partnership,” respectively. The coefficient loadings are shown in Table 1 and Figure 1.

**Table 1:** Rotated factor loadings for the contesting orientations scale—Chinese version (*n* = 116).

**Partnership orientation**		**War orientation**	
**Item**	**Factor loading**	**Item**	**Factor loading**
6 The purpose of competition is to bring out the best in everyone 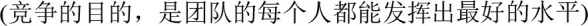	0.84	11 In sports, like in war, opponents stand between you and success 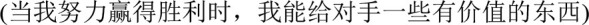	0.85
8 When I try hard to win, I am giving something of value to my opponent 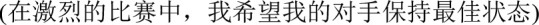	0.81	7 Competition is war 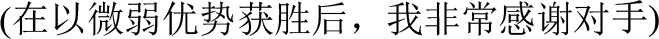	0.83
3 In tight contests, I want my opponents to be at their best 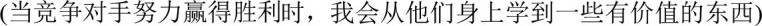	0.77	10 Sport is battling against opponents 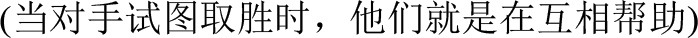	0.83
12 After a narrow win, I really appreciate my opponents 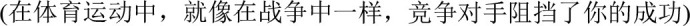	0.73	9 Sport is a fight to see who is best 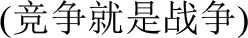	0.80
2 When my opponents try hard to win, they are giving me something of value 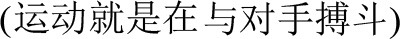	0.72	4 When I compete, my opponent is my enemy 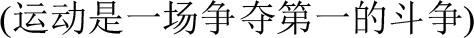	0.73
5 When opponents try to win, they are helping each other 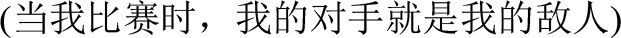	0.69	1 In sport, the goal is to conquer your opponent 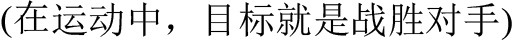	0.69

### 3.3 Confirmatory factor analysis

We used the software Amos 26.0 to conduct confirmatory factor analysis on the formal measured samples. The results showed that the fitting indexes met the statistical indicators, χ2 = 108.67, df = 53, RMSEA = 0.07, NFI = 0.91, CFI = 0.95, GFI = 0.93, PGFI = 0.63. The model fits well and has good structural validity.

### 3.4 Validity assessment

By unifying the classification of the “College Students Competitive Psychology Scale” and using the Pearson correlation analysis method, the relationship between the average score of the College Students' Competitive Psychology Scale and the average score of the scale was explored, and it was found that there is a significant positive relationship between the two. There is a direct relationship (*r* = 0.38, *P* < 0.01). The correlation coefficient between the scores of each dimension of COS and the total score of PPCSCS is 0.61–0.81 (*P* < 0.01). The calibration validity of COS and PPCSCS meets the standards.

### 3.5 Internal consistency reliability

The total Cronbach's α coefficient of the COS scale is 0.79, and the Cronbach's α coefficients of the partnership metaphor and war metaphor subscales are 0.85 and 0.88, respectively, with good internal consistency. The Cronbach's α of the scale changes after deleting one of the items. The results show that no significant change in Cronbach's α was found after deleting one of the items, indicating that the items of the scale have good discrimination. See Tables 2, 3 for details.

**Table 2:** Internal consistency reliability and test-retest reliability.

**Project**	**Cronbach′s α (*n* = 227)**	**Test-retest reliability (*n* = 150)**
COS	0.79	0.78
Partnership orientation	0.85	0.83
War orientation	0.88	0.86

**Table 3:** Changes in the Cronbach′s α coefficient of the total scale after deleting each item.

**Item**	**Cronbach^′^s α after deleting items**
1	0.78
2	0.79
3	0.79
4	0.77
5	0.79
6	0.79
7	0.76
8	0.79
9	0.76
10	0.75
11	0.76
12	0.79

### 3.6 Split-half reliability

As shown in Table 4, the correlation coefficient between the first and second parts of the Chinese version of the competitive orientation scale is 0.57, and the two parts are highly correlated. The Guttman split-half reliability coefficient is 0.71, and the split-half reliability is good.

**Table 4:** Split-half reliability.

**Cronbach^′^s α**	**Part 1**	**Value**	**0.57**

		Number of items	6^a^
	Part 2	Value	0.75
		Number of items	6^b^
	Total number		12.00
Correlation coefficient			0.58
Spearman-Brown			0.73
Guttman half coefficient			0.71

^a^A1, A2, A3, A4, A5, A6.

^b^A7, A8, A9, A10, A11, A12.

### 3.7 Test-retest reliability

The retest data after 1 month showed that the correlation coefficients of the total scale and each dimension were between 0.78 and 0.86, of which the retest reliability of the COS total scale was 0.78, and the retest reliability (r) of the two subscales was 0.83 and 0.86, respectively. See Table 2 for details.

## 4 Discussion

The choice of competitive orientation of athletes not only affects the performance of athletes at a competitive level, but also predicts the personality of college athletes. However, there is currently no measurement method for competitive orientation in China. This study intends to verify the applicability of the Competitive Orientation Scale (COS) among college athletes in Chinese universities by conducting a questionnaire survey on college athletes in Jiangsu Province. On this basis, the scale was translated, and exploratory factor analysis, reliability and validity tests, and confirmatory factor analysis were conducted. The results show that the model adaptation results of this study are in good agreement, and its reliability and validity are also high.

From the results of exploratory factor analysis and item analysis, the commonality of the 12 items is statistically significant, so the 12 items of the original scale can be retained, showing a stable 2-factor structure. According to the results of exploratory factor analysis and item analysis, it can be seen that the commonality of the 12 items is statistically significant, so the 12 items of the original scale can be retained, and a stable 2-factor structure is shown, namely: partnership metaphor and war metaphor. The internal consistency coefficients of the overall and two dimensions of the competitive orientation scale are both above 0.79, and its reliability is good. After deleting one of the items, no significant change in Cronbach's α was found, indicating that the items of the scale have good discrimination. The split-half reliability analysis of the scale found that the correlation coefficient between the first and second parts of the Chinese version of the competitive orientation scale was 0.57, and the two parts were highly correlated. The Guttman split-half reliability coefficient was 0.71, and the split-half reliability was good.

In addition, this study also found that the mean score of the competitive orientation scale and its subscales were positively correlated with the scores of the calibrated scale. This is consistent with previous research results. For example, individuals with good cooperative and competitive personalities know how to cooperate with others in a timely manner to engage in good competition (Zhang, 2011). Through the analysis of the mediating effect of competitive attitude, it was found that achievement goal-oriented and mastery avoidance goal-oriented people rarely adopt a positive competitive attitude, so their mental health level is relatively low (Sheng et al., 2015). By retesting 150 formal subjects, the retest reliability of COS was 0.78, and the retest reliability of the two components was 0.83 and 0.86, indicating that the scale has good stability.

In summary, the competitive orientation scale has high reliability and validity in the cultural context of college athletes in Jiangsu Province, China. However, the subjects selected in this study were college athletes in Jiangsu Province, and there are certain limitations in applicability. It is worth mentioning that age is a negative predictor of war tendency. Older athletes do not like war metaphors (Choresh, 2015). In the future, more research on age can be added. Each finding can pave the way for further research and theoretical development. Future research should expand the scope of sampling, conduct sampling in multiple regions, multiple age groups, and multiple sports, and adopt longitudinal tracking. Verify the applicability of the Chinese version of COS in a larger scope and promote it.

## Data Availability

The raw data supporting the conclusions of this article will be made available by the authors, without undue reservation.
